# Right ventricular free wall longitudinal strain and strain rate quantification with cardiovascular magnetic resonance based tissue tracking

**DOI:** 10.1007/s10554-020-01895-5

**Published:** 2020-05-27

**Authors:** Yang-Yang Qu, Hao Li, Wolfgang Rottbauer, Gen-Shan Ma, Dominik Buckert, Volker Rasche

**Affiliations:** 1 Internal Medicine II, Ulm University Medical Center, Ulm, Germany; 2 Medical School of Southeast University, Nanjing, China; 3 Department of Cardiology, Zhongda Hospital, Southeast University, Nanjing, China

**Keywords:** Cardiovascular magnetic resonance, Tissue tracking, Right ventricle, Strain, Strain rate

## Abstract

Cardiovascular magnetic resonance based tissue tracking (CMR-TT) was reported to provide detailed insight into left ventricular mechanical features. However, inadequate knowledge of the right ventricle (RV) mechanical deformation has been acquired by this advanced technique so far. It was the aim of this study to establish reference values of RV free wall (RVFW) global, regional and segmental longitudinal peak strain and strain rate (LS and LSR), and to investigate the gender- and age-related difference as well as the base-to-apex gradient of RVFW-LS and LSR with CMR-TT. 150 healthy volunteers (75 males/females) were retrospectively and continuously recruited and subdivided into three age groups (G_20–40_, G_41–60_ and G_61–80_). RVFW global, regional (basal, middle-cavity and apical) and segmental LS (GLS, RLS, SLS) along with systolic and diastolic LSR were generated by post-hoc CMR-TT analysis of standard steady-state free precession long-axis four-chamber view cine images acquired at 1.5T field strength. The reference value of myocardial RVFW-GLS was − 24.9 ± 5.2%. We found that females showed more negative GLS than males except in the youngest group, and no age-related difference of GLS was observed in both gender groups. RLS and SLS presented with the same age-related tendency as GLS. The basal and middle-cavity LS were similar between each other and significantly larger than apical LS. RVFW-GLSR resulted as − 1.73 ± 0.58 s^−1^ and 1.69 ± 0.65 s^−1^ during systolic and diastolic phases, respectively. The diastolic GLSR of males tended to decline with the ageing and was significantly lower than that of females in G_61–80_ group. Regional and segmental LSR showed significant gender-related differences in certain basal and apical region/segments without any age-related effects. CMR-TT overcomes the difficulty in measuring RV global and segmental deformation. The establishment of the vendor-, gender- and segment-specific reference values of RVFW-LS and LSR is essential for the rapid and efficient utilization of CMR-TT modality in the clinical routine.

## Background

There is strong evidence that impairment of right ventricular (RV) function plays an important role in developing myocardial diseases [1, 2], including cardiomyopathies, and congenital, ischemic, valvular and pulmonary heart diseases [3–5]. The quantification of RV function has increasingly gained clinical interest and relevance over recent years. RV measurements as such are challenging due to its complex anatomical shape, thin trabeculated myocardium, significant load dependence, and variability of filling with respiration.

Cardiovascular magnetic resonance (CMR) has proven promising for providing high spatial and temporal resolution data [6] of the RV without the known limitations of echocardiography introduced by the RV retrosternal position and thin myocardium (3–5 mm) [7, 8]. Traditionally, RV ejection fraction (EF) derived from the volumetric changes of the RV between end-diastole (EDV) and end-systole (ESV) phases is regarded as a powerful and independent parameter to evaluate RV global contractile function, but cannot provide details on global or regional myocardial deformation. Myocardial strain analysis has been reported as a sensitive surrogate to EF to detect subclinical alterations of myocardial function [9, 10], with remarkable diagnostic and prognostic value in heart failure, pulmonary hypertension and ischemic heart disease [5, 8, 11, 12].

CMR based tissue tracking (CMR-TT) enables the post-hoc offline analysis of dynamic deformation and derives mechanical global and regional parameters including strain and strain rate (SR) over the whole cardiac cycle from conventional cine data [13–16]. Strain analysis has extensively be evaluated for the left ventricle (LV) and reference values have be published for different analysis tools [17, 18]. Application of the technique to the RV is still limited [19, 20] and further vendor-specific reference values are required before wide utilization in clinical routine.

It was the objective of this study to investigate gender- and age-specific reference values of RV free wall (RVFW) global, regional and segmental transmural longitudinal peak strain (LS) and strain rate (LSR) in 150 healthy subjects.

## Methods

### Study population

CMR data of 150 patients (mean age 49.8 ± 17.3 years) not suffering from cardiovascular diseases were included in this retrospective analysis. Inclusion criteria were: above 18 years of age, LVEF ≥ 55%, RVEF ≥ 45%, with no history of cardiovascular or pulmonary diseases or related risk factors (e.g. coronary artery diseases, cardiac arrhythmia, pulmonary dysfunction, hypertension, dyslipidemia, smoking, diabetes mellitus or impaired glucose tolerance).

To investigate gender- and age-related differences of RVFW-LS and LSR, the enrolled subjects were selected equally into gender groups and subdivided into three age groups as G_20–40_ (age range 20–40 years), G_41–60_ (age range 41–60 years) and G_61-80_ (age range 61–80 years).

This study was approved by the local ethics committee. All participants provided written informed consent.

### CMR protocol

All participants underwent a conventional CMR examination on a clinical 1.5T whole-body MRI system (Achieva 1.5T, Philips Healthcare, Best, The Netherlands) at Ulm University Medical Center. All data were acquired with a cardiac 32-channel phased-array receive coil. A stack of short-axis cine images covering the entire left and right ventricle and long-axis cine images (2, 3, 4-chamber) were acquired applying an electrocardiogram-gated steady-state free precession (SSFP) breathhold sequence. Typical acquisition parameters were as: echo time TE = 1.5 ms, repetition time TR = 3.0 ms, flip angle = 55°, spatial resolution = 1.7 mm × 1.7 mm, field of view FOV = 360 mm × 325 mm, slice thickness s_D_ = 8 mm, no slice gap, and 32 cardiac phases.

Biventricular morphological and functional assessment was performed by two experienced CMR physicians with a standard commercial software package (ViewForum®, Philips Healthcare, Best, The Netherlands). All RV volumetric parameters were normalized to the body surface area [BSA (m^2^) = 0.007184 ×  height^0.725^(cm) × weight^0.425^(kg)] to minimize the impact of body size. RV end-diastolic volume index (RVEDVI), RV end-systolic volume index (RVESVI) and RV stroke volume index (RVSVI) and RVEF were assessed to quantify RV morphologic and functional properties. DICOM images were exported to an external workstation for subsequent CMR-TT analysis.

### CMR-TT analysis

RVFW-LS and LSR were derived from the RV long-axis four-chamber view using the commercial specialized software CVI^42^ (version 5.6.3, Circle, Calgary, Canada) [21, 22] by an experienced user of the package.

**Fig. 1:**
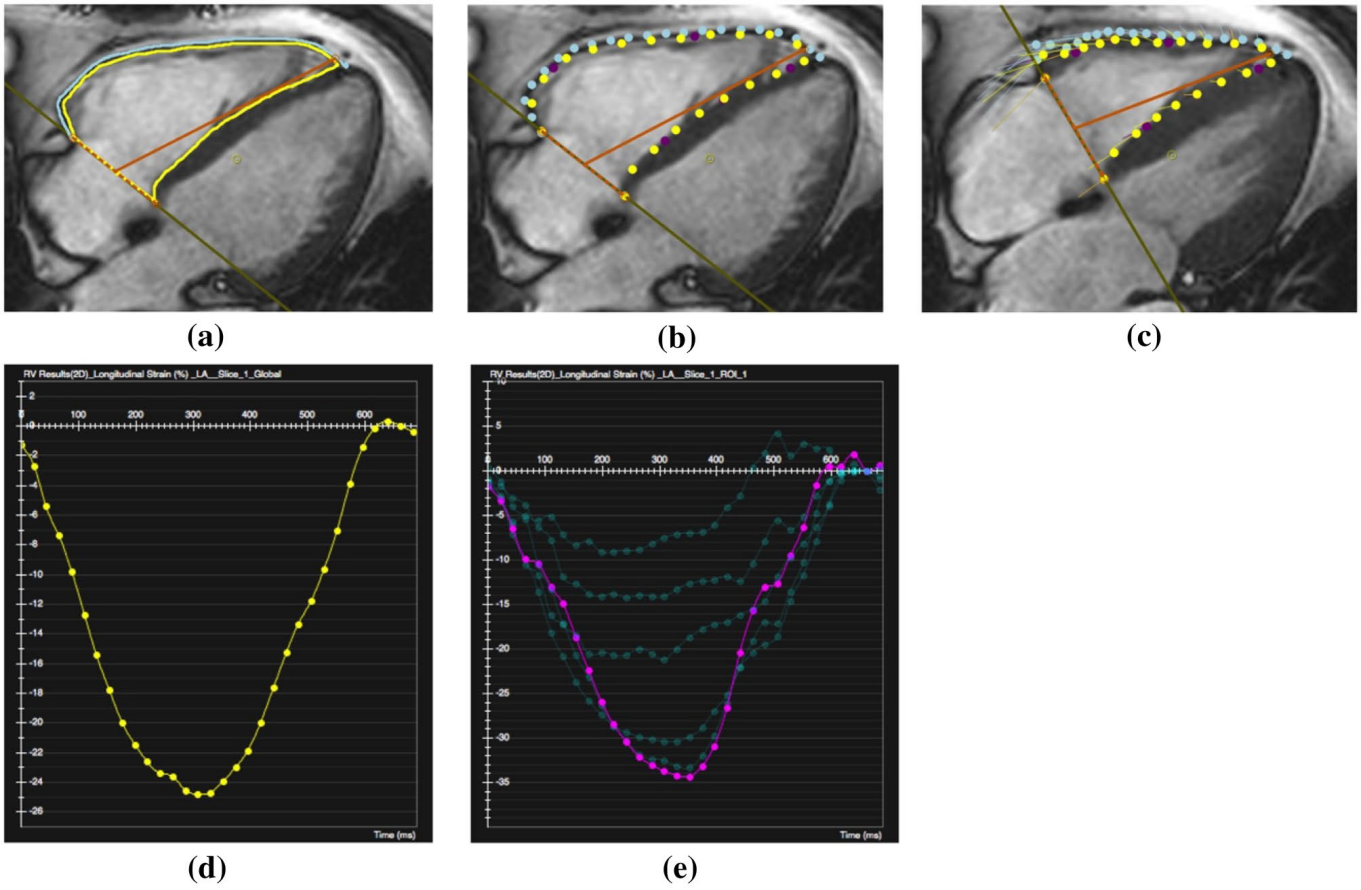
CMR-TT analysis and RVFW time-LS curves. **a** Mark tricuspid annular plane and apex with an orange T bar, and delineate RV endocardium and RVFW epicardium with yellow and blue lines at end diastole. **b** The yellow endocardial and blue epicardial boundary points are presented at the phase next to end diastole. **c** The positions of boundary points are shown at end systole. The yellow or blue round points represent the final positions of myocardial feature patterns, and the tiny starting points of connected lines indicate the initial positions of feature patterns at end diastole. **d** The time-strain curve of RVFW-GLS. **e** The time-strain curves of RVFW-SLS

For segmentation, the RV tricuspid annular plane and apex were marked, followed by the manual delineation of the RV endocardial border and FW epicardial border at end diastole (Fig. 1a). Subsequently, the software automatically propagated and traced myocardial features phase to phase throughout the whole cardiac cycle. In cases of inaccurate tracking of the RV border, manual corrections of delineation were performed.

From the tracked tissue features, CVI^42^ was used to generate dynamic mechanical information of global and segmental two-dimensional RV strain for all cardiac phases. Global as well as segmental (6 segments with the same length of RVFW at end diastole) LS and LSR were computed via the Lagrangian formulas as: $LS_{t} = \left( {L_{t} - L_{0} } \right)/L_{0} ,LSR_{t} = dLS_{t} /dt$, respectively [13]. ${L_{t} }$ refers to the final length of the cardiac tissue, and $L_{0}$ represents the initial length at end diastole. Peak values as well as LS and LSR curves of the global myocardium (GLS and GLSR) and segments (SLS and SLSR) were generated. The peak value of time-strain and time-SR curves (Fig. 1d, e) corresponded with the data generated automatically in the text report. Regional LS and LSR were calculated by averaging the respective two segmental values within the corresponding region.

### Statistical analysis

Continuous variables are expressed as mean value with standard deviation (SD). Gaussian distribution was tested applying a Shapiro–Wilk test, and extreme outliers (0/150 to 9/150 for each parameter) were excluded. The statistical significance between two groups or parameters were assessed with a two-tailed Student’s *t* test or a Mann–Whitney *U* test, as appropriate. Age-related difference among three groups was investigated using analysis of variance (ANOVA) or Kruskal–Wallis test followed by Bonferroni correction, as applicable. Correlation between age and RVFW-GLS was analyzed with a Spearman correlation coefficient (r). Comparisons among basal, middle-cavity and apical LS or LSR were tested with Friedman test. Statistical significance was assumed with *P* < 0.05. Intra- and inter-observer variability of RVFW global, regional and segment LS and LSR were performed for fifteen randomly selected subjects using Bland–Altman analysis. Intra-class correlation coefficient (ICC) and coefficient of variation (CoV) were used to assess the reproducibility and variability. All analyses were performed with IBM SPSS 24.0 and Medcalc 19.1.

## Results

### Baseline characteristics of the study participants

The detailed baseline characteristics of the subgroups are presented in Table 1. Average age resulted as 49.8 ± 17.3 years old, without a significant difference between males and females in each age group. Compared with females, males showed significantly larger BSA, RVEDVI, RVESVI and RVSVI, as well as lower RVEF (*P* < 0.05). This tendency got more and more pronounced with increasing age. In the G_61–80_ group, RVEF of females resulted 12.9% higher than in males (69.2 ± 6.3% vs. 61.3 ± 5.6%, *P* < 0.05). Furthermore, age-related decline of heart rate (HR) was detected in males, and decrease of RV morphological characters as well as increase of RVEF were observed in females. For females in the G_61-80_ group, RVEDVI reduced by 24.0% in comparison with the G_20–40_ group (61.6 ± 9.4 mL/m^2^ vs. 81.0 ± 12.4 mL/m^2^, *P* < 0.05).

**Table 1:** Baseline characteristics of the healthy volunteers

	G_20-40_		G_41–60_		G_61–80_		Males	Females	All
	Males	Females	Males	Females	Males	Females	(n = 75)	(n = 75)	(n = 150)
Baseline demographics									
Age (years)	29.5 ± 5.5	29.4 ± 7.4	50.8 ± 5.7	51.2 ± 6.5	69.3 ± 6.1	68.8 ± 5.7	49.9 ± 17.3^†^	49.8 ± 17.5^†^	49.8 ± 17.3
BSA (m^2^)	2.0 ± 0.2	1.7 ± 0.1*	2.1 ± 0.2	1.9 ± 0.2*	2.0 ± 0.2	1.8 ± 0.1*	2.0 ± 0.2	1.8 ± 0.2*^†^	1.9 ± 0.2
BMI (kg/m^2^)	24.9 ± 3.6	23.8 ± 6.6*	26.4 ± 3.9	28.1 ± 5.9	25.6 ± 3.8	25.7 ± 4.5	25.6 ± 3.8	25.8 ± 5.9^†^	25.7 ± 4.9
HR (bpm)	74.0 ± 18.7	71.6 ± 11.5	66.3 ± 16.0	70.9 ± 14.6	63.8 ± 14.1	68.6 ± 11.2	68.0 ± 16.7^†^	70.4 ± 12.4	69.2 ± 14.7
RV morphology and contractile function									
RVEDVI (mL/m^2^)	88.9 ± 16.0	81.0 ± 12.4	83.1 ± 17.5	72.7 ± 11.2*	78.9 ± 12.5	61.6 ± 9.4*	83.6 ± 15.8	71.8 ± 13.6*^†^	77.7 ± 15.8
RVESVI (mL/m^2^)	38.5 ± 9.9	31.1 ± 6.3*	31.8 ± 10.5	25.3 ± 7.7*	30.9 ± 8.0	20.4 ± 4.5*	33.7 ± 10.0^†^	25.8 ± 7.6*^†^	29.7 ± 9.7
RVSVI (mL/m^2^)	51.1 ± 8.9	49.9 ± 7.9	51.3 ± 9.1	47.4 ± 6.8	48.2 ± 7.0	42.2 ± 5.1*	50.2 ± 8.4	46.5 ± 7.4*^†^	48.4 ± 8.1
RVEF (%)	58.6 ± 8.0	61.7 ± 4.1*	62.5 ± 6.5	65.6 ± 7.0	61.3 ± 5.6	69.2 ± 6.3*	60.8 ± 6.9	65.5 ± 6.6*^†^	63.2 ± 7.2

Results are reported as Mean ± SD

*BSA* body surface area, *BMI* body mass index, *HR* heart rate, *RVEDVI* right ventricular end-diastolic volume index, *RVESVI* right ventricular end-systolic volume index, *RVSVI* right ventricular stroke volume index, *RVEF* right ventricular ejection fraction

*P < 0.05: males vs. females. ^†^*P* < 0.05: age-related difference among three age groups

### RVFW global, regional and segmental LS

**Fig. 2:**
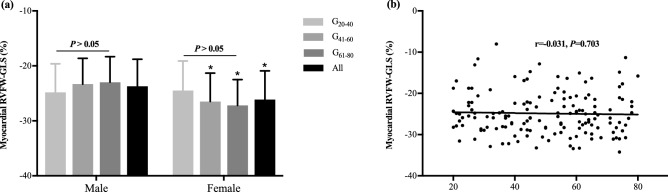
The reference values of RVFW-GLS and its gender- and age-related differences. **a** Gender- and age-related difference of RVFW-GLS. Gender-related difference of RVFW-GLS was found in G_41–60_ and G_61–80_ groups, and age-associated difference wasn’t detected in each gender subgroup. *P < 0.05: significant difference between males and females in the same age subgroup. **b** The correlation between age and RVFW-GLS. There was no correlation between age and RVFW-GLS since Spearman correlation coefficient (r) resulted as − 0.031 (*P* = 0.703)

RVFW global, regional and segmental LS (GLS, RLS and SLS) derived from CMR-TT are provided in Table 2. The reference value of GLS was − 24.9 ± 5.2%. Females showed higher amplitude of GLS than males in the groups G_41–60_ and G_61–80_ (*P* < 0.05). With the increase of age, males presented with smaller GLS, which was opposite to females. However, no significant age-related difference of GLS was noticed in both gender groups (*P* > 0.05) and no correlation was observed between age and GLS (r = − 0.031, *P* = 0.703, Fig. 2).

**Table 2:** RVFW-GLS, RLS and SLS

	G_20–40_		G_41–60_		G_61–80_		Male	Female	All
	Males	Females	Males	Females	Males	Females	(n = 75)	(n = 75)	(n = 150)
RVFW-GLS (%)	− 24.8 ± 5.2	− 24.5 ± 5.4	− 23.3 ± 4.7	− 26.5 ± 5.2*	− 22.8 ± 4.9	− 27.2 ± 4.7*	− 23.7 ± 4.9	− 26.1 ± 5.2*	− 24.9 ± 5.2
RVFW-RLS (%)									
Basal	− 28.1 ± 7.4	− 25.9 ± 9.8	− 27.9 ± 5.9	− 27.4 ± 6.1	− 24.6 ± 6.6	− 29.1 ± 6.2*	− 26.8 ± 6.8	− 27.5 ± 7.6	− 27.1 ± 7.2
Middle-cavity	− 26.2 ± 5.2	− 26.0 ± 5.7	− 25.8 ± 4.0	− 28.9 ± 4.0*	− 26.5 ± 5.1	− 29.5 ± 4.0*	− 26.2 ± 4.8	− 28.1 ± 4.8*	− 27.2 ± 4.9
Apical	− 18.1 ± 5.4	− 22.8 ± 7.1*	− 17.8 ± 5.4	− 21.8 ± 8.5	− 17.3 ± 5.4	− 21.2 ± 5.8*	− 17.8 ± 5.4	− 21.9 ± 7.1*	− 19.8 ± 6.6
RVFW-SLS (%)									
Segment 1	− 27.2 ± 8.7	− 24.5 ± 12.9	− 27.2 ± 7.6	− 26.9 ± 7.2	− 23.0 ± 7.7	− 28.4 ± 8.4*	− 25.8 ± 8.2	− 26.6 ± 9.8	− 26.2 ± 9.0
Segment 2	− 29.0 ± 6.7	− 27.3 ± 8.5	− 28.5 ± 5.3	− 27.8 ± 6.1	− 26.2 ± 6.5	− 29.7 ± 4.7	− 27.9 ± 6.2	− 28.3 ± 6.6	− 28.1 ± 6.4
Segment 3	− 27.5 ± 6.0	− 27.0 ± 6.4	− 28.0 ± 4.5	− 29.2 ± 5.6	− 27.2 ± 6.2	− 30.7 ± 4.5*	− 27.6 ± 5.5	− 29.0 ± 5.7*	− 28.3 ± 5.6
Segment 4	− 24.9 ± 5.6	− 25.1 ± 6.8	− 23.6 ± 5.2	− 28.5 ± 4.2*	− 25.1 ± 6.0	− 28.3 ± 4.2	− 24.5 ± 5.5	− 27.3 ± 5.4*	− 25.9 ± 5.6
Segment 5	− 22.7 ± 5.1	− 24.7 ± 6.0	− 21.4 ± 4.5	− 25.2 ± 6.7*	− 20.3 ± 5.7	− 24.9 ± 5.3*	− 21.5 ± 5.1	− 24.9 ± 5.9*	− 23.2 ± 5.8
Segment 6	− 13.6 ± 7.3	− 20.9 ± 10.1*	− 14.3 ± 8.6	− 18.4 ± 11.3	− 14.3 ± 5.9	− 17.5 ± 7.7	− 14.1 ± 7.2	− 18.9 ± 9.8*	− 16.5 ± 8.9

Results are reported as Mean ± SD

*RVFW* right ventricular free wall, *GLS* global longitudinal peak strain, *RLS* regional longitudinal peak strain, *SLS* segmental longitudinal peak strain

*P < 0.05: males vs. females

Among the whole population, the LS at middle-cavity and apical regions and segments presented gender-related but no age-related differences. A base-to-apex gradient of RLS was observed. The basal and middle-cavity LS were similar (*P* > 0.05) but significantly larger than apical LS (*P* < 0.05).

### RVFW global, regional and segmental LSR

CMR-TT allows the measurement of RVFW global, regional and segmental LSR (GLSR, RLSR and SLSR) throughout the whole cardiac cycle, the mean and SD of systolic and diastolic peak SR are presented in Tables 3 and 4.

**Table 3:** RVFW systolic GLSR, RLSR and SLSR

	G_20–40_		G_41–60_		G_61–80_		Male	Female	All
	Males	Females	Males	Females	Males	Females	(n = 75)	(n = 75)	(n = 150)
RVFW-GLSRs (s^−1^)	− 1.67 ± 0.67	− 1.57 ± 0.54	− 1.63 ± 0.43	− 1.89 ± 0.60	− 1.73 ± 0.57	− 1.88 ± 0.58	− 1.68 ± 0.56	− 1.78 ± 0.59	− 1.73 ± 0.58
RVFW-RLSRs (s^−1^)									
Basal	− 2.86 ± 0.91	− 2.44 ± 0.84	− 3.00 ± 1.01	− 2.86 ± 1.01	− 3.00 ± 1.07	− 2.88 ± 0.85	− 2.95 ± 0.99	− 2.73 ± 0.91	− 2.84 ± 0.95
Middle-cavity	− 2.78 ± 0.98	− 2.59 ± 0.80	− 2.56 ± 0.57	− 2.92 ± 0.89	− 2.81 ± 1.04	− 3.04 ± 1.04	− 2.72 ± 0.88	− 2.85 ± 0.92	− 2.79 ± 0.90
Apical	− 2.00 ± 0.61	− 2.27 ± 0.72	− 2.08 ± 0.85	− 2.17 ± 0.64	− 1.92 ± 0.64	− 2.06 ± 0.74	− 2.00 ± 0.70	− 2.16 ± 0.69	− 2.08 ± 0.70
RVFW-SLSRs (s^−1^)									
Segment 1	− 3.30 ± 1.64	− 2.37 ± 1.18	− 3.17 ± 1.39	− 2.99 ± 1.33	− 3.26 ± 1.69	− 3.10 ± 1.21	− 3.24 ± 1.56	− 2.82 ± 1.26	− 3.03 ± 1.43
Segment 2	− 2.75 ± 1.18	− 2.51 ± 0.85	− 2.85 ± 0.87	− 2.79 ± 1.00	− 2.77 ± 0.85	− 2.67 ± 0.85	− 2.79 ± 0.97	− 2.66 ± 0.90	− 2.72 ± 0.93
Segment 3	− 2.81 ± 1.04	− 2.59 ± 0.84	− 2.69 ± 0.90	− 2.90 ± 1.07	− 3.04 ± 1.35	− 3.26 ± 1.27	− 2.84 ± 1.11	− 2.92 ± 1.09	− 2.88 ± 1.10
Segment 4	− 2.76 ± 1.09	− 2.60 ± 0.99	− 2.44 ± 0.79	− 2.93 ± 1.02	− 2.74 ± 1.11	− 2.83 ± 0.92	− 2.64 ± 1.00	− 2.79 ± 0.98	− 2.71 ± 0.99
Segment 5	− 2.56 ± 1.11	− 2.60 ± 1.00	− 2.61 ± 1.17	− 2.43 ± 0.72	− 2.34 ± 0.93	− 2.36 ± 0.88	− 2.50 ± 1.06	− 2.46 ± 0.87	− 2.48 ± 0.97
Segment 6	− 1.75 ± 1.01	− 1.99 ± 0.85	− 1.62 ± 0.95	− 2.00 ± 0.98	− 1.51 ± 0.67	− 1.79 ± 0.92	− 1.63 ± 0.88	− 1.92 ± 0.91	− 1.77 ± 0.91

Results are reported as Mean ± SD

*RVFW* right ventricular free wall, *GLSRs* global longitudinal systolic peak strain rate, *RLSRs* regional longitudinal systolic peak strain rate, *SLSRs* segmental longitudinal systolic peak strain rate

**Table 4:** RVFW diastolic GLSR, RLSR and SLSR

	G_20–40_		G_41–60_		G_61–80_		Male	Female	All
	Males	Females	Males	Females	Males	Females	(n = 75)	(n = 75)	(n = 150)
RVFW-GLSRd (s^−1^)	1.81 ± 0.61	1.67 ± 0.55	1.50 ± 0.59	1.80 ± 0.72	1.46 ± 0.61	1.91 ± 0.73*	1.59 ± 0.62^†^	1.80 ± 0.67*	1.69 ± 0.65
RVFW-RLSRd (s^−1^)									
Basal	3.10 ± 1.14	2.53 ± 1.03	3.16 ± 1.39	2.96 ± 1.20	3.13 ± 1.21	3.23 ± 0.91	3.13 ± 1.23	2.91 ± 1.08	3.14 ± 1.37
Middle-cavity	2.38 ± 0.90	2.54 ± 0.94	2.35 ± 0.85	2.58 ± 0.82	2.46 ± 0.99	2.41 ± 0.66	2.40 ± 0.90	2.51 ± 0.81	2.51 ± 0.97
Apical	2.03 ± 0.79	2.21 ± 0.82	1.90 ± 0.75	2.10 ± 0.69	1.84 ± 0.70	2.05 ± 0.79	1.92 ± 0.74	2.12 ± 0.76	2.16 ± 0.99
RVFW-SLSRd (s^−1^)									
Segment 1	3.90 ± 1.99	2.81 ± 1.61	3.82 ± 2.04	3.78 ± 2.37	3.88 ± 1.96	3.77 ± 1.45	3.87 ± 1.97	3.46 ± 1.89	3.66 ± 1.93
Segment 2	2.43 ± 0.59	2.35 ± 0.96	2.51 ± 1.11	2.39 ± 0.88	2.45 ± 0.90	2.69 ± 0.95	2.46 ± 0.88	2.48 ± 0.93	2.47 ± 0.90
Segment 3	2.53 ± 1.03	2.58 ± 1.22	2.30 ± 1.11	2.53 ± 0.93	2.34 ± 0.90	2.49 ± 0.73	2.39 ± 1.01	2.54 ± 0.97	2.46 ± 0.99
Segment 4	2.35 ± 1.07	2.50 ± 0.87	2.40 ± 1.04	2.64 ± 1.04	2.58 ± 1.22	2.38 ± 0.82	2.45 ± 1.10	2.51 ± 0.91	2.48 ± 1.00
Segment 5	2.10 ± 0.96	2.46 ± 0.76	2.28 ± 1.10	2.60 ± 0.96	2.09 ± 0.92	2.44 ± 0.80	2.16 ± 0.99	2.50 ± 0.84*	2.33 ± 0.93
Segment 6	1.95 ± 1.00	2.02 ± 1.07	1.51 ± 0.78	1.84 ± 1.00	1.60 ± 0.66	1.73 ± 1.00	1.69 ± 0.84	1.86 ± 1.01	1.77 ± 0.92

Results are reported as Mean ± SD

*RVFW* right ventricular free wall, *GLSRd* global longitudinal diastolic peak strain rate, *RLSRd* regional longitudinal diastolic peak strain rate*, SLSRd* segmental longitudinal diastolic peak strain rate

*P < 0.05: males vs. females

^†^*P* < 0.05: age-related difference among three age groups

The systolic GLSR was − 1.68 ± 0.56 s^−1^ for males and − 1.78 ± 0.59 s^−1^ for females (*P* > 0.05). No significant gender-related difference of systolic RLSR and SLSR was observed. No significant age-related difference was found for all LSR at systole. Unlike systolic GLSR, diastolic GLSR of males was significantly lower than that of females as 1.59 ± 0.62 s^−1^ vs. 1.80 ± 0.67 s^−1^ (*P* < 0.05). Similar to diastolic GLSR, a significant gender-related difference of diastolic SLSR was observed in the segment 5. With the increase of age, diastolic GLSR significantly decreased in males, which is opposite to the trend observed in females.

A base-to-apex gradient of both systolic and diastolic RLS was also observed, which was characterized with the largest RLS value in the base and the smallest RLS value in the apex (*P* < 0.05).

### Intra- and inter-observer reproducibility of RVFW-LS and LSR

**Fig. 3:**
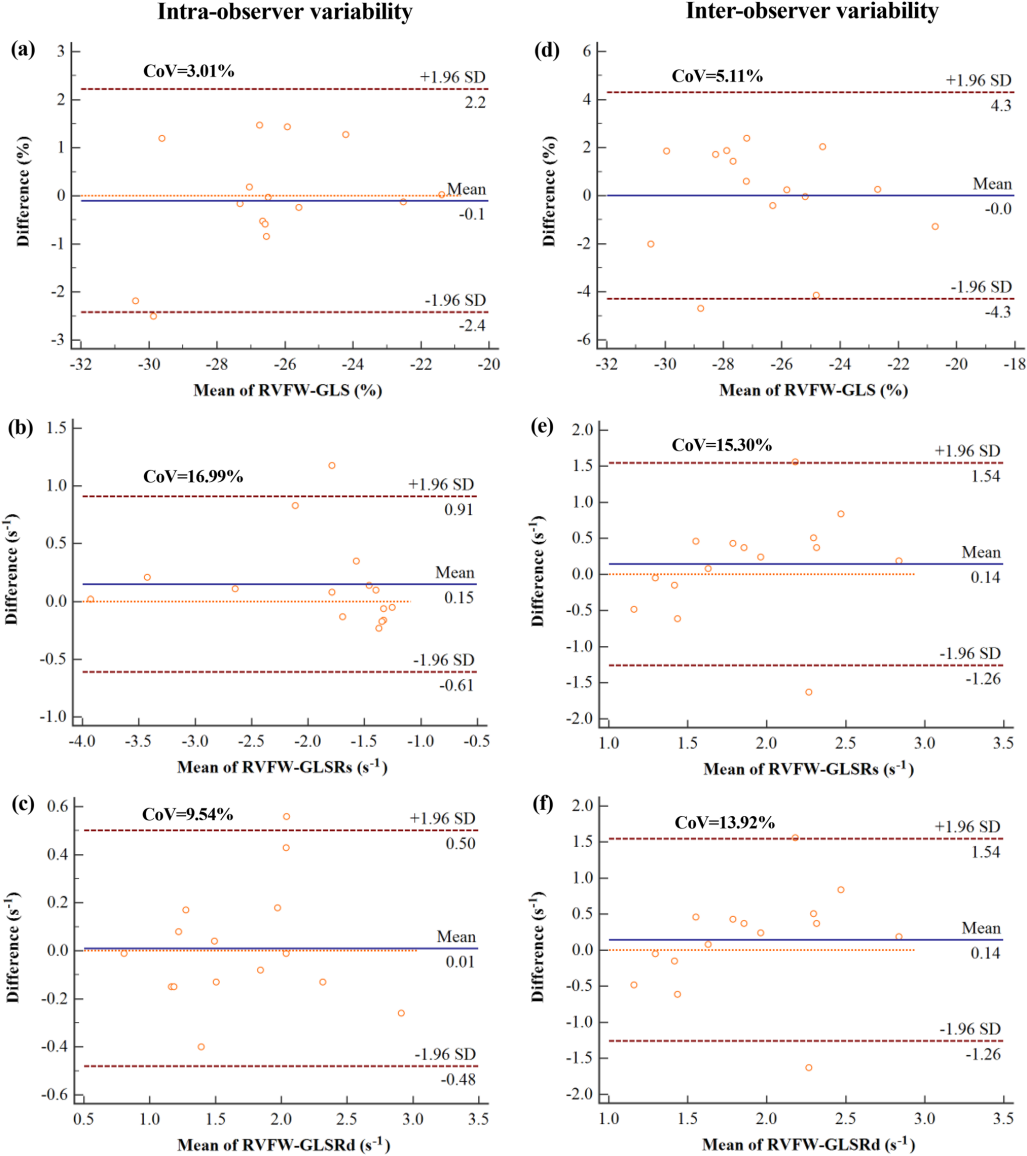
Bland–Altman plots showing intra- and inter-observer variability of RVFW-GLS and RVFW-GLSR

The intra- and inter-observer variability of RVFW global, regional and segmental LS and LSR are presented in Fig. 3 and Tables 5, 6 and 7. Bland–Altman analysis showed that the global evaluation of RVFW-LS revealed the best intra- and inter-observer reproducibility (CoV ≤ 5.11%) compared with that of systolic and diastolic RVFW-GLSR (CoV ranging from 9.54% to 16.99%). The CoV of regional and segmental LS was no more than 11.52% except for the most apical segment 6 (CoV = 18.24% and 29.65% for intra- and inter-observer assessment, respectively). The variability for SLSR was in the range of 10.41–29.61% and 15.11–31.83% for intra- and inter-observer assessment, respectively.

**Table 5:** Intra- and inter-observer variability of RVFW-LS

	Intra-observer variability			Inter-observer variability		
	MD ± SD	ICC (95% CI)	CoV	MD ± SD	ICC (95% CI)	CoV
RVFW-GLS	0.85 ± 0.80	0.94 (0.83–0.98)	3.01	1.66 ± 1.35	0.83 (0.48–0.94)	5.11
RVFW-RLS						
Basal	1.82 ± 1.74	0.89 (0.67–0.96)	5.88	1.92 ± 1.46	0.90 (0.71–0.97)	4.88
Middle-cavity	1.64 ± 1.32	0.95 (0.84–0.98)	4.89	1.75 ± 1.95	0.94 (0.82–0.98)	7.18
Apical	2.10 ± 1.37	0.84 (0.54–0.95)	7.43	2.89 ± 1.90	0.72 (0.17–0.91)	10.45
RVFW-SLS						
Segment 1	2.48 ± 2.35	0.90 (0.71–0.97)	8.01	2.24 ± 1.39	0.93 (0.80–0.98)	4.66
Segment 2	1.48 ± 1.65	0.87 (0.60–0.96)	5.55	1.93 ± 1.88	0.84 (0.52–0.95)	6.28
Segment 3	1.34 ± 0.86	0.97 (0.90–0.99)	3.00	1.72 ± 1.62	0.95 (0.84–0.98)	5.57
Segment 4	2.48 ± 1.89	0.90 (0.71–0.97)	7.53	2.61 ± 2.69	0.88 (0.66–0.96)	10.70
Segment 5	1.66 ± 1.20	0.90 (0.69–0.97)	5.12	2.73 ± 2.67	0.55 (− 0.35–0.85)	11.52
Segment 6	2.95 ± 2.46	0.83 (0.50–0.94)	18.24	4.09 ± 3.88	0.66 (− 0.01–0.89)	29.65

*MD* mean difference, *SD* standard deviation, *ICC* intra-class correlation coefficient, *CI* confidence interval, *CoV* coefficient of variation. The unit of CoV is %

**Table 6:** Intra- and inter-observer variability of systolic RVFW-LSR

	Intra-observer variability			Inter-observer variability		
	MD ± SD	ICC (95% CI)	CoV	MD ± SD	ICC (95% CI)	CoV
RVFW-GLSRs	0.25 ± 0.32	0.94 (0.83–0.98)	16.99	0.30 ± 0.28	0.94 (0.82–0.98)	15.30
RVFW-RLSRs						
Basal	0.36 ± 0.50	0.94 (0.82–0.98)	15.65	0.70 ± 0.59	0.75 (0.26–0.92)	19.13
Middle-cavity	0.37 ± 0.31	0.92 (0.76–0.97)	11.47	0.41 ± 0.38	0.92 (0.75–0.97)	13.98
Apical	0.35 ± 0.39	0.91 (0.74–0.97)	18.62	0.49 ± 0.35	0.83 (0.49–0.94)	16.13
RVFW-SLSRs						
Segment 1	0.65 ± 0.76	0.91 (0.74–0.97)	20.96	1.07 ± 0.96	0.62 (− 0.12–0.87)	27.91
Segment 2	0.48 ± 0.51	0.92 (0.76–0.97)	18.06	0.72 ± 0.54	0.79 (0.37–0.93)	19.99
Segment 3	0.54 ± 0.46	0.87 (0.61–0.96)	16.76	0.56 ± 0.49	0.90 (0.69–0.97)	17.24
Segment 4	0.40 ± 0.42	0.89 (0.67–0.97)	15.85	0.47 ± 0.42	0.89 (0.67–0.97)	15.78
Segment 5	0.53 ± 0.56	0.83 (0.49–0.94)	22.91	0.80 ± 0.70	0.61 (− 0.15–0.87)	27.99
Segment 6	0.44 ± 0.36	0.79 (0.33–0.93)	23.31	0.58 ± 0.39	0.62 (− 0.17–0.88)	23.28

*GLSRs* global longitudinal systolic peak strain rate, *RLSRs* regional longitudinal systolic peak strain rate, *SLSRs* segmental longitudinal systolic peak strain rate, *MD* mean difference, *SD* standard deviation, *ICC* intra-class correlation coefficient, *CI* confidence interval, *CoV* coefficient of variation. The unit of CoV is %

**Table 7:** Intra- and inter-observer variability of diastolic RVFW-LSR

	Intra-observer variability			Inter-observer variability		
	MD ± SD	ICC (95% CI)	CoV	MD ± SD	ICC (95% CI)	CoV
RVFW-GLSRd	0.18 ± 0.16	0.95 (0.84–0.98)	9.54	0.22 ± 0.23	0.91 (0.73–0.97)	13.92
RVFW-RLSRd						
Basal	0.44 ± 0.35	0.91 (0.72–0.97)	10.62	0.54 ± 0.39	0.75 (0.26–0.92)	11.70
Middle-cavity	0.38 ± 0.24	0.79 (0.36–0.93)	10.64	0.38 ± 0.41	0.92 (0.75–0.97)	18.18
Apical	0.38 ± 0.30	0.85 (0.54–0.95)	16.07	0.53 ± 0.48	0.83 (0.49–0.94)	25.39
RVFW-SLSRd						
Segment 1	0.72 ± 0.68	0.92 (0.75–0.97)	15.86	0.85 ± 0.62	0.92 (0.77–0.97)	15.11
Segment 2	0.34 ± 0.25	0.88 (0.63–0.96)	10.41	0.53 ± 0.48	0.79 (0.36–0.93)	19.00
Segment 3	0.51 ± 0.58	0.48 (− 0.54–0.83)	23.72	0.53 ± 0.53	0.52 (− 0.42–0.84)	22.89
Segment 4	0.60 ± 0.36	0.65 (− 0.04–0.88)	17.32	0.59 ± 0.47	0.68 (0.04–0.89)	21.12
Segment 5	0.40 ± 0.38	0.89 (0.67–0.96)	17.40	0.71 ± 0.54	0.45 (− 0.63–0.82)	24.55
Segment 6	0.48 ± 0.47	0.68 (0.06–0.89)	29.61	0.54 ± 0.51	0.56 (− 0.30–0.85)	31.83

*GLSRd* global longitudinal diastolic peak strain rate, *RLSRd* regional longitudinal diastolic peak strain rate*, SLSRd* segmental longitudinal diastolic peak strain rate, *MD* mean difference, *SD* standard deviation, *ICC* intra-class correlation coefficient, *CI* confidence interval, *CoV* coefficient of variation. The unit of CoV is %

## Discussion

This study investigated RVFW-LS and systolic and diastolic LSR at global, regional and segmental levels with CMR-TT in 150 healthy volunteers without cardiovascular and pulmonary diseases. The major findings of this study are as follows: (i) CMR-TT reproducibly provides RVFW-LS and LSR by semi-automatic tracking of RV myocardial tissue features over the cardiac cycle, (ii) females presented with significantly higher amplitude of RVFW-LS and diastolic LSR than males, especially in the elderly group, (iii) even though neither an age-related difference nor a correlation between age and RVFW-GLS was observed, diastolic RVFW-GLSR in general showed a trend to decrease in males with ageing, and (iv) a base-to-apex gradient in RV longitudinal shortening was observed with almost similar contraction in basal and middle location and minimum contraction at the apical segment.

### The measurement of RVFW-LS and LSR with CMR-TT

Recent improvements in cardiac imaging have made the measurement of RV function and deformation feasible and practical in clinical routine. It has proven to be useful for the early detection of RV functional and mechanical abnormalities before obvious changes in the EF. However, conventional echocardiography, as the most available and cheapest imaging modality for evaluating RV structure and function, is limited due to the insufficient image quality (narrow acoustic windows into RV) and subjective quantification [7, 22]. Overcoming the limitations of echocardiography, CMR has emerged as the gold standard due to its high spatial and temporal resolution, good soft tissue contrast without ionizing radiation.

CMR-TT has been developed to quantify dynamic deformation of the myocardium. CMR-TT is based on conventional SSFP cine images and does not require any additional measurements as in tissue tagging [11]. Deformation information is derived from automatic tracking of tissue features over the cardiac cycle [13] and has presented as sensitive and convenient offline post-processing analysis [23].

Myocardial strain is defined as the relative lengthening and thickening (known as positive strain) or shortening and thinning (known as negative strain) of myocardial fibers compared with the end-diastolic length. Strain is the most frequently used index to estimate myocardial deformation. The strain rate (SR), represented as the derivative of the strain, quantifies the degree of change in myocardial deformation with respect to time [13]. Strain and SR can be applied to analyze myocardial deformation at global and segmental levels. Both parameters have been reported to be relatively independent on loading conditions and to identify active contraction [24], and as such to be advantageous in comparison to conventional volumetric parameters such as EF and stroke volume.

The longitudinal shortening is the major contribution to RV contraction, and RV function was shown highly dependent on longitudinal shortening [25]. LS and LSR may identify subclinical RV dysfunction before the development of abnormalities reflected by RVEF or stroke volume (as similar as in LV) [11]. Considering that interventricular septum is mainly a constituent part of the LV and only contributes 20% to RV systolic performance [26], analysis of the RVFW appears superior to quantify the RV contraction.

RVFW-GLS was reported to be the most accurate functional marker correlating with the degree of RV myocardial fibrosis in patients with advanced HF [26]. It also been shown as a prognostic marker in acute non-massive pulmonary embolism with area under the curve (AUC) as 0.754 [27]. In addition, intraoperative RV functional decline was found to be primarily related with deterioration of RVFW deformation rather than that of the interventricular septum [28]. Further RVFW-GLS has been reported to provide important diagnostic information in patients with depressed RVEF (AUC = 0.918) [29], RV pressure overload (AUC = 0.95) [30], and proximal right coronary artery lesion-induced ischemic heart disease (AUC = 0.79) [31].

Even though RVFW-LS and LSR have indicated to be clinical relevant parameters, age- and gender-specific reference values for global an segmental analysis are rare. In this contribution, respective reference values for CMR-TT were derived from four-chamber long-axis SSFP cine images with a strong focus on the free wall.

### Reproducibility of RVFW-LS and LSR

Liu et al. derived a mean RVFW-GLS of − 24.2 ± 3.59% among 100 normal healthy subjects, which was in good concordance with our result of − 24.9 ± 5.1%. The systolic RVFW-GLSR was as − 1.54 ± 0.41 s^−1^, which was close to the data we obtained [19]. The RVFW is thinner and thus more potential to present higher longitudinal deformation against pulmonary resistance compared to the interventricular septum [32]. Hence the reference values of RVFW-GLS derived from the RVFW only couldn’t be simply utilized to estimate the mechanical deformation of the whole RV myocardium. Further, the reference values aren’t interchangeable for other modalities or vendors using different algorithms. For example, the average value of RVFW-LS acquired by Mararu et al. using 2-dimensional speckle-tracking echocardiography was − 30.5 ± 3.9%, which was far from ours [33].

It’s a consensus that strain measurement is more reliable than SR, and global evaluation is superior to segmental assessment. This observation was confirmed by our intra- and inter-observer reproducibility assessment. The poorest reproducibility of SLS and SLSR in apical segments may limit the regional application of CMR-TT technique in measuring RVFW apical deformation. The lower intra- and inter-observer variability of RVFW-GLS found in ours and others’ researches [21, 34] clearly indicate the use of the global parameter as a reliable and reproducible clinical and research parameter.

### Gender- and age-related differences of RVFW-LS and LSR

Gender-related differences of RVFW-LS and LSR have been discussed before. Truong et al. analyzed the RVFW-GLS and SLS among 50 patients (4–81 years old) without known cardiac pathology with the same software package. He demonstrated that the GLS was comparable between males and females as − 22.22 ± 3.4% and − 22.80 ± 3.5%, respectively [22]. Liu et al. also reported no significant differences with values of − 23.9 ± 3.59% for males and − 24.6 ± 3.59% for females (*P* = 0.34) [35]. However, our study reports a significant higher amplitude of RVFW-GLS in females compared with that in males as − 26.1 ± 5.2% vs. − 23.7 ± 4.9% (*P* < 0.05) on the basis of a larger sample size. Our finding is consistent with previous studies including the largest echocardiographic speckle-tracking study performed among 276 healthy volunteer by Muraru et al. [22, 33]. We hypothesized that the gender-related difference of GLS was probably caused by the different RV size and systolic function, since males presented with higher RVEDVI, RVESVI and RESVI, and lower RVEF. The systolic LSR was comparable between males and females (*P* > 0.05), which implies the similar rate of myocardial shortening during systole. The RVFW diastolic LSR is regarded as a marker of RV relaxation, which may reduce with collagen deposition, fibrosis and impaired calcium uptake of cardiomyocytes [36]. Females presented higher diastolic GLSR compared with males as 1.80 ± 0.67% vs. 1.59 ± 0.62% (*P* < 0.05), implying the better compliance of RVFW. Meanwhile, the decrease of diastolic GLSR in males demonstrated the deterioration of diastolic function of RVFW with ageing. In contrast, the absence of a significant decrease in the diastolic LSR in females might be explained by the low feasibility of SR assessment.

With the potential age-related increase in pulmonary artery pressure, pulmonary vascular resistance, cardiomyocyte loss and subsequent replacement by fibrosis, RVFW mechanical function was supposed to decrease with aging [36]. We did not find any age-related difference of RVFW-GLS and systolic GLSR in neither gender groups and no correlation between age and RVFW-GLS or systolic GLSR. This finding was in line with the finding of previous studies [22, 33, 37]. This finding may simplify the establishment of reference values and clinical use of these two parameters. However, normative data from multicenter and larger size of population are still necessary for the further validation.

### Segmental variability of RVFW-SLS and SLSR

The RVFW base-to-apex gradient was measured and resulted as − 27.1 ± 7.2%, − 27.2 ± 4.9% and − 19.8 ± 6.6% for SLS, and − 2.84 ± 0.95%, − 2.79 ± 0.90% and − 2.08 ± 0.70% for systolic SLSR, respectively (*P* < 0.05). RVFW-SLS and systolic SLSR reached the lowest values in the apical territories and became comparable between the basal and mid-cavity regions. Our outcome is in good accordance the finding from a meta-analysis of 226 healthy children (< 21 years of age) from 10 studies [38] and others’ researches [32, 39]. Abnormality of this physiological principle of base-to-apex heterogeneity may differentiate the early alteration of RV myocardial contractility or afterload among patients with various pathological changes. Furthermore, even though the reproducibility of segmental strain and SR are lower than global parameters [40], the awareness of segmental reference values and alterations contributes to the better understanding and localization of impaired myocardium.

## Study limitation

There were several limitation to this study. Firstly, this is a single-center study among a limited number (n = 150) of healthy volunteers. Even though the number of healthy subjects enrolled in this study exceeds most previous studies, investigations in multiple centers with larger sample size are still necessary to further clarify the intrinsic principle of RVFW-LS and LSR. Secondly, it’s challenging to quantify RV myocardial deformation due to the thin wall and crescent shape. Hence, we merely focused on the deformation in the longitudinal direction with long-axis four-chamber view cine images, which theoretically ensured the good feasibility and reproducibility. Manual adjustment and visual inspection by an experienced physicians were performed during the measurement, which ensured the quality of operation. Thirdly, the reference values may be vendor-specific and likely do not apply with other vendors.

## Conclusion

In conclusion, our study provides reference values of RVFW-LS and LSR for specific vendor CVI^42^ using CMR-TT technique. Meanwhile, the gender- and age-related differences and base-to-apex gradient of RVFW-LS and LSR were investigated among a relatively large size of healthy population. The vendor-, gender- and segment-specific reference values we established with CMR-TT modality contribute to the standard incorporation of RVFW global and segmental LS and LSR into the clinical routine.

## Data Availability

The datasets used and/or analyzed during the current study are available from the corresponding author on reasonable request.

## Abbreviations

ANOVA: Analysis of variance
AUC: Area under the curve
BMI: Body mass index
BSA: Body surface area
CI: Confidence interval
CMR: Cardiovascular magnetic resonance
CMR-TT: Cardiovascular magnetic resonance based tissue tracking
CoV: Coefficent of variation
EF: Ejection fraction
FOV: Field of view
GLS: Global longitudinal peak strain
GLSR: Global longitudinal peak strain rate
HR: Heart rate
ICC: Intra-class correlation coefficient
LV: Left ventricle/left ventricular
r: Correlation coefficient
RLS: Regional longitudinal peak strain
RLSR: Regional longitudinal peak strain rate
RV: Right ventricle/right ventricular
RVEDVI: Right ventricular end-diastolic volume index
RVESVI: Right ventricular end-systolic volume index
RVFW: Right ventricular free wall
SLS: Segmental longitudinal peak strain
SLSR: Segmental longitudinal peak strain rate
SSFP: Steady-state free precession
SVI: Stroke volume index
TE: Echo time
TR: Repetition time
